# Type IVA Choledochal Cyst: Is Hepatic Resection Necessary?

**DOI:** 10.1155/1996/17026

**Published:** 1996

**Authors:** Russell Strong

**Affiliations:** Princess Alexandra Hospital Ipswich Road Woolloongabba Queensland 4102 Australia

## Abstract

*Background*: This study concerns patients who have choledochal cyst with intrahepatic and extrahepatic involvement (type IVA cyst). The extent of excision and the necessity of hepatectomy, including the intrahepatic cyst in these patients have not been clarified.

*Study design*: We have performed excision of the extrahepatic cyst with hepaticojejunostomy upon 13 patients with type IVA cyst during a 16 year period. The present study was done to examine the size of the anastomotic opening by direct cholangiography two weeks postoperatively. The long-term results were assessed to find the appropriate operative management for patients with type IVA cysts.

*Results*: Intrahepatic cysts were present in both hepatic lobes in 11 patients (85 percent). None of the patients had carcinoma after excision of extrahepatic cyst during the follow-up period, which ranged from two months to 16 years. Postoperative late complications occurred in three patients (23 percent), hepatolithiasis in two and cholangitis in one. The anastomotic opening of hepaticojejunostomy was 13.3±4.5 mm in diameter two weeks postoperatively, which was not significantly different when compared with that in ten patients without late complications (13.4±4.9 mm). The late complications were successfully treated with either antibiotics or percutaneous transhepatic cholangioscopy, and none required a reoperation.

*Conclusions*: The results suggest that additional hepatectomy is not required because carcinoma has rarely occurred from the intrahepatic cyst. Excision of an extrahepatic cyst with a wide hepaticojejunostomy is an acceptable operative management for patients with type IVA cysts.


any patients developing HCC over the course of at least several decades would be more cost effective.

In conclusion, the article is humbling. In their overtly honest and unpretentious evaluation of the meager clinical data available regarding the malignant transformation of LCAs, Foster and Berman clearly confront us with our limited knowledge of LCAs. Despite the paucity of data, they have proposed tentative management guidelines which are reasonable with the proviso that the benefit-risk ratio of operation to observation is overwhelmingly patient favorable. Perhaps our response in appreciation to these authors should be the establishment of a world-wide registry of patients with histologically-proven LCAs to determine their course; only collectively will we answer the questions which they so humbly pose.

David M Nagorney
Department of Surgery
Mayo Clinic
200 First Street Southwest
Rochester
Minnesota 55905
United States of America

### TYPE IVA CHOLEDOCHAL CYST: IS HEPATIC RESECTION NECESSARY?

### ABSTRACT

*Chijiiwa, K., Komura, M. and Kameoka, N. (1994) Postoperative followup of patients with type IVA choledochal cysts after excision of extrahepatic cyst. Journal of The American College of Surgeons; 179: 641–645.*

***Background:*** **This study concerns patients who have choledochal cyst with intrahepatic and extrahepatic involvement (type IVA cyst). The extent of excision and the necessity of hepatectomy, including the intrahepatic cyst in these patients have not been clarified.**
***Study design:*** **We have performed excision of the extrahepatic cyst with hepaticojejunostomy upon 13 patients with type IVA cyst during a 16 year period. The present study was done to examine the size of the anastomotic opening by direct cholangiography two weeks postoperatively. The long-term results were assessed to find the appropriate operative management for patients with type IVA cysts.**
***Results:*** **Intrahepatic cysts were present in both hepatic lobes in 11 patients (85 percent). None of the patients had carcinoma after excision of extrahepatic cyst during the follow-up period, which ranged from two months to 16 years. Postoperative late complications occurred in three patients (23 percent), hepatolithiasis in two and cholangitis in one. The anastomotic opening of hepaticojejunostomy was 13.3±4.5 mm in diameter two weeks postoperatively, which was not significantly different when compared with that in ten patients without late complications (13.4±4.9 mm). The late complications were successfully treated with either antibiotics or percutaneous transhepatic cholangioscopy, and none required a reoperation.**
***Conclusions:*** **The results suggest that additional hepatectomy is not required because carcinoma has rarely occurred from the intrahepatic cyst. Excision of an extrahepatic cyst with a wide hepaticojejunostomy is an acceptable operative management for patients with type IVA cysts. *J. Am. Coll. Surg.*, 1994, 179: 641–645.**

KEY WORDS: Choledochal cyst hepatic resection.

### PAPER DISCUSSION

The paper by Chijiiwa *et al.*, reviewed the long term results of surgical treatment of patients with type IV A choledochal cysts. The type I-solitary extrahepatic cyst and type IV A-extrahepatic and intrahepatic cysts, comprise the great proportion of choledochal cysts. There is widespread acceptance that the treatment of the type I cyst is surgical excision with a Roux-en-Y biliary reconstruction to the confluence of the hepatic ducts. This eliminates the biliary stasis and recurrent cholangitis related to the extrahepatic abnormality and removes the major site of susceptibility to malignancy, which is so difficult to diagnose before it is beyond surgical cure. The authors endeavoured to clarify whether surgical resection of the extrahepatic component of the type IV A cystic disease was sufficient to obtain a good long term outcome, similar to that achieved following excision of a type I choledochal cyst.

They performed resection of the extrahepatic cyst with hepatojejunostomy in 13 patients and the follow-up ranged from two months to 16 years. They measured the size of the hepatojejunostomy two weeks postoperatively and found no significant difference in the size of the anastomosis between those who developed hepatolithiasis and cholangitis (23%) and those without such complications. There is no doubt that there is a requirement to construct an anastomosis of adequate size without subsequent stricture formation but this does not eliminate the propensity for the later development of biliary complications. Biliary sludge formation and intraductal lithiasis can result from stagnation of bile in the saccular dilatation of the intrahepatic ducts [1] and it must be expected that this complication has the potential to occur in the retained cystic disease in the liver. This was confirmed by the authors of this paper when they found no anastomotic stricture in their patients who developed hepatolithiasis and cholangitis.

The authors quoted case reports of carcinoma arising in the retained intraheptic bile ducts but none of their patients developed a carcinoma in the follow-up period. They surmised that the elimination of regurgitation of pancreatic juice into the biliary tree could be a factor. Benhidjeb[2] noted that malignancy in association with choledochal cyst was not always in the cyst and not totally prevented by cyst excision. However, the majority of reported cases occurred in the dilated intrahepatic ducts and not those that were of normal size. Pancreatic juice is not the only potential carcinogenic agent. Stasis, chronic inflammation and hepatolithiasis are also known to be associated with biliary malignancy. The biliary epithelium in the retained intrahepatic cysts is somewhat unstable and with the appropriate trigger, is prone to malignant transformation. The mutagenicity of bile acids such as deoxycholate and lithocholate could be implicated.

The mean follow-up of the 13 patients was 6.08 years. A longer period is probably necessary before concluding that excision of the extra-hepatic cyst is sufficient to prevent carcinoma occurring in the retained intrahepatic cyst disease. The reviewer has witnessed one patient who developed carcinoma in the intrahepatic biliary cyst, 25 years after excision of the extrahepatic portion of a type IV A choledochal cyst. It is possible that cholangiocarcinoma complicating retained intrahepatic cysts of type IV A has been under-reported. As many of the patients are relatively young at the time of the initial surgery, they will have a lifespan of many decades with Damocles Sword hovering over them.

The aim of surgery should be the complete excision of the cystic disease. In type IV A, this is only possible in about 15-20% of cases where the intrahepatic component is unilobar. Here, hepatic resection of the diseased intrahepatic portion is combined with excision of the extrahepatic choledochal cyst and Roux-en-Y hepatojejunostomy to the remaining hepatic duct. It is obviously not practical to resect the intrahepatic disease when it is bilobar. At the time of resection of the extrahepatic cyst and hepatojejunostomy, dilatation of any intrahepatic strictures should be performed. If severe disease predominates in one portion of bilobar disease, this should be resected. Two of the 13 cases in the series were treated in this manner. Total hepatectomy and liver transplantation is indicated when cirrhosis and/or portal hypertension is present or when recurrent and persistent hepatolithiasis and cholangitis complicates satisfactory extrahepatic excision and hepatojejunostomy.

Most authors advocate close follow-up but there is no defined policy on how this should be performed. Malignant disease is usually diagnosed at an advanced stage on the basis of clinical and radiological evaluation. The early detection of malignant transformation will probably require the development of tumour markers which would allow surveillance to become more scientifically based.

Professor Russell Strong
Director of Surgery
Princess Alexandra Hospital
Ipswich Road
Woolloongabba
Queensland
Australia 4102

### REFERENCES

1. Desmet, V.J. (1992) Congenital diseases of intrahepatic bile ducts: variations on the theme "ductal plate malformation". *Hepatology*, **16**: 1069–1083.
2. Benhidjeb, B., Munster, B., Ridwelski, K., Rudolph, B., Mau, H., Lippert, H. (1994) Cystic dilatation of the common bile duct: surgical treatment and long term results. *British Journal of Surgery*, **81**: 433–436.

